# Knowledge, attitude and practice of lifestyle modification recommended for hypertension management and the associated factors among adult hypertensive patients in Harar, Eastern Ethiopia

**DOI:** 10.1177/2050312120953291

**Published:** 2020-09-01

**Authors:** Saron Bogale, Kirubel Minsamo Mishore, Assefa Tola, Abraham Nigussie Mekuria, Yohanes Ayele

**Affiliations:** 1 Department of Clinical Pharmacy, School of Pharmacy, College of Health and Medical Sciences, Haramaya University, Harar, Ethiopia; 2 Department of Epidemiology and Biostatistics, School of Public Health, College of Health and Medical Sciences, Haramaya University, Harar, Ethiopia; 3 Department of Pharmacology, School of Pharmacy, College of Health and Medical Sciences, Haramaya University, Harar, Ethiopia

**Keywords:** Lifestyle modification, hypertension, knowledge, attitude, practice

## Abstract

**Introduction:**

Hypertension is an overwhelming global challenge. Appropriate lifestyle modifications are the cornerstone for the prevention and control of hypertension. In this regard, lack of knowledge and poor attitude toward lifestyle modification have been a major setback.

**Objective:**

To assess knowledge, attitude and practice of lifestyle modification recommended for hypertension management and the associated factors among adult hypertensive patients in Harar, Eastern Ethiopia.

**Methods:**

Hospital-based cross-sectional study was conducted among 274 hypertensive patients in Hiwot Fana Specialized University Hospital, from 1 March to 30 May 2019. The pre-tested structured questionnaire was used, and the data were collected through an interview. The data were analyzed using SPSS version 20. A multivariate logistic regression model was fitted to determine independent predictors of knowledge and practice of lifestyle modifications among hypertensive patients. Adjusted odds ratio (AOR) at 95% confidence interval (CI) was used for predicting the independent effect of each variable on the outcome variables.

**Results:**

From the total participants, 200 (73.0%) of participants had good knowledge, 182 (66.4%) had favorable attitude and 136 (49.6%) had good practice on lifestyle modification recommended for hypertension management. Regarding factors associated with lifestyle modification, being in age range of 46–64 years (AOR: 4.08, 95% CI: 1.14–14.56); having formal education (AOR: 3.93, 95% CI: 1.27–12.23); being government employee (AOR: 8.06, 95% CI: 1.40–46.32) and being housewives (AOR: 5.10, 95% CI: 1.26–20.79) were factors significantly associated with good knowledge of lifestyle modification, However, favorable attitude was found to be the only factor associated with good practice of lifestyle modification (AOR: 9.20, 95% CI: 2.60–32.24).

**Conclusion:**

In the current study, knowledge and attitude toward lifestyle modification recommended for hypertension management was fairly good but practice level was poor. Concerted strategies are required to increase the knowledge, attitude and practice of the lifestyle modification measures in this population group.

## Introduction

Arterial hypertension, also known as high or raised blood pressure (BP), is a global public health issue. It contributes to the burden of heart disease, stroke and kidney failure, hence to premature mortality and disability.^1^ According to World Health Organization (WHO) 2013 report, globally cardiovascular disease accounts for approximately 17 million deaths a year, nearly one-third of the total. Of these, hypertension-related complications account for 9.4 million deaths worldwide every year.^1^ Developing countries share significant burden as they are experiencing epidemiological transition from communicable to non-communicable chronic diseases.^2^ For example, in Africa, Ethiopia included, 15% of the population is thought to have hypertension.^3,4^ The prevalence of hypertension was found to be high among adults aged ⩾50 years, ranging from 22.3% to 90.0%.^5^

The factors contributing to the hypertension are mainly environmental, genetic and behavioral factors such as smoking, high alcohol intake and high fat intake. These risk factors can be counteracted through lifestyle modification (LSM).^6–9^ According to WHO, increment in prevalence of hypertension and other cardiovascular disease in developing countries has been associated with aging of the populations, urbanization and socioeconomic changes favoring sedentary habits.^1^

Hypertension can be adequately managed through drug treatment as well as lifestyle changes. LSM, in addition to lowering BP, can also reduce other cardiovascular risk factors.^10^ Furthermore, the involved cost is minimal and there are hardly any risks. Hypertensive patients irrespective of their disease stage are encouraged to adopt these measures.^11,12^ Unawareness of LSM and failure to apply it was one of the identified patient-related barriers to BP control.^13^

In hypertensive individuals, LSM can be recommended as an initial treatment before starting drug therapy or as an adjuvant. LSM has proven role in hypertension management. For example, according to Whelton et al.,^14^ LSM can decrease systolic blood pressure (SBP) approximately by 11 mmHg. Moreover, LSM may enable drug step-down and drug withdrawal ultimately when the control is deemed adequate, particularly in patients who are highly committed to maintain lifestyle changes.

Different types of LSMs are indicated for hypertension management, including weight loss for obese patients, sodium reduction not exceeding 1.5 g daily, alcohol intake reduction, smoking cessation, avoidance of excessive caffeine, diet modification such as low intake of saturated and total fat and increase in fruits and vegetables intake.^15,16^ Despite its proven effect, the implementation of LSM is often the overlooked part of hypertension management. One problem for lack of LSM among hypertensive patient is lack of awareness and poor practice. For example, a study conducted in Ghana showed mean knowledge score on LSM of 2.5 (standard deviation (SD): 1.25) out of 5^17^ whereas in Nigeria only 33 out of 104 (31.7%) had good knowledge.^18^ Similarly, the study participant displayed poor attitude and adherence toward LSM in both study.

Various both modifiable and non-modifiable factors have been associated with poor implementation of LSM. Age, educational status, monthly income, years since diagnosis and co-morbidity were among factors significantly associated with LSM practice.^19^ Gender and educational background were associated with knowledge of LSM.^17^ It is important to identify modifiable factors and work toward improving it in order to adequately control hypertension and prevent its sequel. The few existing studies conducted in Ethiopia indicated inconsistent data.^19–21^ For instance, the level of good practice to LSM was found to be 23% in Addis Ababa^21^ whereas it was found to be 61.39% in Bishoftu.^20^ As our knowledge goes, there is no published study conducted in the study setting. In this study, we examined KAP toward LSM recommended for hypertension management and factors associated with knowledge and practice of LSM among adult hypertensive patients on chronic care unit Hiwot Fana Specialized University Hospital (HFSUH).

## Methods and materials

### Study area and period

The study was conducted in HFSUH from 1 March to 30 May 2019. HFSUH is located in Harar City which is found about 525 km from Addis Ababa in eastern part of Ethiopia. HFSUH was established in 1941. It is a referral hospital for population in Harar city and its surrounding. The hospital has different inpatient and outpatient departments. This study was conducted in outpatient chronic care unit of HFSUH.

### Study design

Hospital-based cross-sectional study was conducted to assess the level of knowledge, attitude and practice (KAP) toward LSM recommended for hypertension management among adult hypertensive patients in chronic care unit of HFSUH.

### Population

All adult hypertensive patients who were on follow-up visit in chronic care unit of HFSUH were source population, whereas all adult hypertensive patients visiting the chronic care unit during the study period were study population. All adult hypertensive patients who had follow-up visit in chronic care unit of HFSUH in the study period were included, and those adult hypertensive patients who were newly diagnosed (less than a year) were excluded from the study.

### Sample size and sampling techniques

Sample size was determined using single proportion formula with 95% confidence level, 5% degree of precision and taking the proportion of hypertensive patients who had sufficient knowledge (72.3%), positive attitude (68.32%) and good practice (61.39%) of LSM obtained from a previous study conducted in Bishoftu General Hospital.^20^ We considered the largest sample size, which was 364. Since our source population was less than 10,000, finite population correction was used. Then, adding 5% non-responses on the largest sample size, the final sample size was 292. All consecutive adult hypertensive patients visiting chronic care unit of HFSUH during the study period and who fulfill the inclusion criteria were included for the purpose of the study.

#### Data collection tools and data collection procedure

Data were collected through face-to-face interview using a pre-tested structured questionnaire. The questionnaire was adapted from similar studies and other related documents investigating KAP of LSM recommended for hypertension management.^14,17,19,20,22,23^ It contains all necessary variables regarding socio-demographics characteristics, KAP of LSM of the respondents. Data were collected by two BSc nurses who have had training on the subject matter and experience in data collection.

### Data quality control

The quality of data was assured by pre-testing the questionnaire, training of the data collectors and supervising the data collection process. Well-designed questionnaire of English version was prepared by the investigators and translated in to local languages (*Afaan Oromoo* and *Amharic*). The instrument was pre-tested before the study period on 5% of the sample patients in Jugel Hospital which is located in Harar City, and amendments were made accordingly. The data collectors were given 1 day training concerning interviewing technique and appropriate data recording. Close supervision was made by investigators during the data collection through observation and review of the questionnaires filled and giving feedback to the data collectors.

### Statistical analyses

Data were entered, cleaned and analyzed using SPSS Version 20 for windows. Appropriate descriptive statistics such as mean (with SD), median (with interquartile range (IQR)) and frequencies (with percentages) were used to describe the study population in relation to relevant variables. Bivariate and multivariate analysis with 95% confidence interval (CI) was employed to infer associations between the independent and dependent variables. Binary logistic regression was used to calculate the crude odds ratio (COR) with 95% CI. Each variable was entered into a logistic regression model so as to determine the presence of statistical significant association with the outcome variable. Multicollinearity among selected independent variables was checked through variance inflation factor (VIF), and none was found. All the explanatory variables with a P-value ⩽0.05 in the bivariate analyses were included in the final multivariable logistic model in order to identify the independent predictors of knowledge and practice of LSM. P-value <0.05 was considered statistically significant. Assumption on fitness of goodness of the final model was checked by Hosmer and Lemeshow test and was found fit.

### Ethical consideration

Ethical approval was obtained from School of Pharmacy, College of Health and Medical Sciences, Haramaya University. A legal authorization letter was written from College of Health and Medical Sciences to study setting to get permission before data collection. The data collectors conducted interview after explaining the purpose of the study. Participation was on voluntary basis, and confidentiality was maintained to encourage accurate and honest self-disclosure. The information obtained was made anonymous and de-identified prior to analysis to ensure confidentiality.

### Operational definition

#### Knowledge

In order to measure the level of knowledge toward LSM recommended for hypertension management among hypertensive patient, we used 10 multiple-choice questions. If respondents get the right answer, it was coded as Yes “1” if not it was coded as No “0.” The respondent’s knowledge scores were aggregated and ranged 0–10. Based on the cumulated score, respondents, who scored mean and above, were considered to have “Good knowledge”; while those who score below mean were considered as having “Poor knowledge” toward LSM to manage hypertension.

#### Attitude

To determine the level of attitude, a set of nine statements pertaining to LSM recommended for hypertension management among hypertensive patients were included in the questionnaire, and respondents were asked to record whether they agreed strongly, agreed slightly, had no opinion about the statement under consideration, disagree slightly, disagree strongly (a five-point Likert-type scale). Based on the cumulated score, respondents, who scored median value and above, were considered to have “favorable attitude”; while those who score below median were considered as having “unfavorable attitude” toward LSM.

#### Practice

In order to determine the extent of practice of LSM recommended for hypertension management among hypertensive patient, we used a set of nine multiple-choice questions. If respondents practiced correctly each question, it was coded as Yes “1” if not it was coded as No “0.” The respondent’s practice scores were aggregated and ranged 0–9. Based on the cumulated score, respondents, who scored mean and above, were considered to have “Good practice”; while those who score below mean were considered as having “Poor practice” toward LSM to manage hypertension.

## Results

### Socio-demographic characteristics

A total of 274 hypertensive patients participated in the study with a response rate of 94%. Half of the total respondents were females (138, 50.4%); 158 (57.7%) of the respondents were between ages 46 and 64 years. The mean age of the study participants was 56.36 years (±11.79, SD). Majority (250, 91.2%) of them were currently married; 204 (74.5%) were urban dwellers, 164 (59.9%) had formal education and 104 (38.0%) were government employee. Nearly two-third (63.5%) of the respondents had an average monthly income below 1000.00 Ethiopian Birr (ETB). Almost two-third (63.7%) of the respondents had normal (18.5–24.9) body mass index (BMI). The mean (±SD) BMI of the participants was 24.07 (±3.21) kg/m^2^. All the study participants were on antihypertensive medication (Table 1).

**Table 1.** Socio-demographic characteristics of hypertensive patients at chronic care unit of HFSUH, Harar, Eastern Ethiopia, 2019 (N = 274).

Variable	Category	Frequency	%
Sex	Male	136	49.6
	Female	138	50.4
Age (year)	⩽45	48	17.5
	46–64	158	57.7
	⩾65	68	24.8
Ethnicity	Oromo	114	41.6
	Amhara	124	45.3
	Harari	22	8.0
	Others	14	5.1
Religion	Christian	182	66.4
	Muslim	92	33.6
Marital status	Currently married	250	91.2
	Not married	24	8.8
Area of residence	Urban	204	74.5
	Rural	70	25.5
Educational status	No formal education	110	40.1
	Formal education	164	59.9
Occupation	Farmer	34	12.4
	Government employee	104	38.0
	Merchant	34	12.4
	Housewife	102	37.2
Body mass index (kg/m^2^)	<18.5	10	3.7
	18.5–24.9	172	63.7
	⩾25	88	32.6
Average monthly income (ETB)	⩽1000	174	63.5
	1001–2500	46	16.8
	⩾2500	54	19.7
Duration on follow-up (year)	⩽5	182	66.4
	>5	92	33.6
Duration on antihypertensive medication (year)	⩽5	184	67.2
	>5	90	32.8

HFSUH: Hiwot Fana Specialized University Hospital; ETB: Ethiopian Birr.

### Knowledge of hypertensive patients on LSM

**Figure 1.**
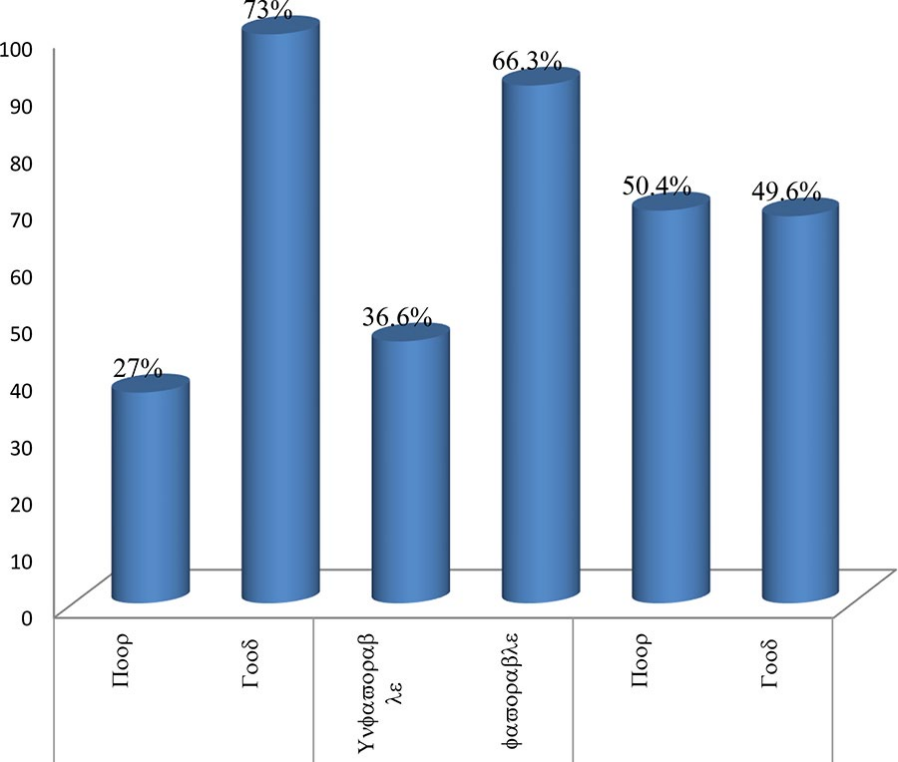
Overall respondents level of knowledge, attitude and practice status of LSM recommended for hypertension management among adult hypertensive patients at chronic care unit of HFSUH, Harar, Eastern Ethiopia, 2019 (N = 274).

A total of 10 knowledge questions were forwarded to assess the respondents’ level of knowledge toward LSM recommended for hypertension management. All 274 (100%) of the respondents were able to recall their recent BP measurement which was further confirmed by cross-checking their follow-up records. Of the respondents, 254 (92.7%) were able to mention at least one LSM recommended for hypertension management. Over two-third (71.5%) of them understood the importance of maintaining normal body weight to controlling their BP. Over three-fourth 78.8%, 77.4% and 78.8% knew the effect of drinking alcohol, smoking cigarette and chewing Khat on controlling BP, respectively (Table 2). Regarding the overall knowledge level of the respondents, 200 (73.0%) participants had good knowledge about LSM recommended for hypertension management (Figure 1).

**Table 2.** Respondents level of knowledge toward LSM recommended for hypertension management among hypertensive patients at chronic care unit of HFSUH, Harar, Eastern Ethiopia, 2019 (N = 274).

Knowledge questions	Yes (%)	No (%)
Knows recent BP measurement	274 (100)	0 (0)
Aware of LSM to manage hypertension	252 (92.0)	22 (8.0)
Can mention at least one LSM to manage hypertension	254 (92.7)	20 (7.3)
Knows the importance of maintaining normal body weight to controlling BP	196 (71.5)	78 (28.5)
Knows the importance of diet in controlling BP	230 (83.9)	44 (16.1)
Know the importance of reducing salt intake in controlling BP	244 (89.1)	30 (10.9)
Know the importance of regular physical activity in controlling BP	244 (89.1)	30 (10.9)
Know the effect of drinking alcohol on controlling BP	216 (78.8)	58 (21.2)
Know the effect of cigarette smoking on controlling BP	212 (77.4)	62 (22.6)
Know the effect of chewing Khat on controlling BP	216 (78.8)	58 (21.2)

LSM: lifestyle modification; HFSUH: Hiwot Fana Specialized University Hospital; BP: blood pressure.

### Attitude of hypertensive patients on LSM

A total of nine attitude assessment questions were forwarded to respondents to assess their level of attitude toward LSM recommended for hypertension management. Majority (89.8%) of the respondents either strongly or slightly agreed on importance of education during their follow-up about LSM recommended for hypertension management. Three-fourth (76.6%) of the respondents either strongly or slightly agreed in importance of maintaining normal body weight to control their BP. Majority (79.6%) of the respondents either strongly or slightly disagree for the question stated as excessive alcohol intake has no effect on BP (Table 3). Regarding the overall attitude score of the respondents, 182 (66.4%) of the participants had favorable attitude toward LSM recommended for hypertension management (Figure 1).

**Table 3.** Respondents level of attitude toward LSM recommended for hypertension management among hypertensive patients at chronic care unit of HFSUH, Harar, Eastern Ethiopia, 2019 (N = 274).

Attitude question	Strongly agree (%)	Agree slightly (%)	Neutral (%)	Disagree slightly (%)	Strongly disagree (%)
Education during follow-up on LSM about hypertension is essential component of HTN management	234 (85.4)	12 (4.4)	28 (10.2)	0 (0)	0 (0)
Regular checking of BP is important part of BP management	256 (93.4)	6 (2.2)	12 (4.4)	0 (0)	0 (0)
Maintaining normal body weight is important for controlling BP	202 (73.7)	8 (2.9)	64 (27.0)	0 (0)	0 (0)
Controlling diet is important for controlling BP	230 (83.9)	6 (2.2)	36 (13.1)	2 (0.73)	0 (0)
Controlling salt intake is important for controlling BP	244 (89.1)	2 (0.73)	28 (10.2)	0 (0)	0 (0)
Regular physical activity is important part of controlling BP	244 (89.1)	4 (1.5)	24 (8.8)	0	1 (0.73)
Excessive alcohol intake has no effect on BP	16 (5.8)	0	40 (14.6)	2 (1.5)	214 (78.1)
Khat chewing has no effect on BP	18 (6.6)	0	52 (19.0)	4 (1.5)	200 (73.0)
Smoking cigarette has no effect on BP	16 (5.8)	0	40 (14.6)	2 (0.73)	216 (78.8)

LSM: lifestyle modification; HFSUH: Hiwot Fana Specialized University Hospital; BP: blood pressure; HTN: hypertension.

### Practice level of hypertensive patients on LSM

A total of nine practice assessment questions were forwarded to respondents to assess their extent of practice toward LSM recommended for hypertension management. Regarding regular checking of BP and weight, almost all (96.4%) of the respondents regularly check their BP and below half (42.3%) check their body weight regularly. Majority (83.2%) of the respondents regularly conduct physical activity for about 30 min/day for most days of the week. Nearly half (42%) of the participants did not plan regularly to include diets rich in fruits, vegetables and low-fat dairy products in meal program. Significant proportion (19%) of hypertensive patients chews Khat (Table 4). Regarding the overall practice level of the respondents, 136 (49.6%) of the participants had good practice of LSM recommended for hypertension management (Figure 1).

**Table 4.** Respondents level of practice toward LSM recommended for hypertension management among hypertensive patients at chronic care unit of HFSUH, Harar, Eastern Ethiopia, 2019 (N = 274).

Practice question	Yes (%)	No (%)
Check BP regularly	264 (96.4)	10 (3.6)
Check weight regularly	116 (42.3)	158 (57.7)
Regularly plan to include diet rich in fruits, vegetables and low-fat dairy products in meal program	158 (57.7)	116 (42.3)
Regularly eat diet rich with high cholesterol^a^	42 (15.3)	232 (84.7)
Regularly consume salt free or low salt diet	222 (81.0)	52 (19.0)
Regularly drink alcohol^a^	22 (8.0)	252 (92.0)
Conduct physical activity regularly (at least 30 min/day, most days of the week)	228 (83.2)	46 (16.8)
Chew Khat^a^	52 (19.0)	222 (81.0)
Smoke cigarette^a^	12 (4.4)	262 (95.6)

LSM: lifestyle modification; HFSUH: Hiwot Fana Specialized University Hospital; BP: blood pressure.

a For these practice questions, those who responded “Yes” were given “0” and responded “No” were given “1” for the purpose of analysis.

### Factors associated with knowledge and practice of LSM

In multivariate logistic regression analysis, age, educational status and occupation of the respondents showed a significant association with knowledge of LSM. Those respondents in age range of 46–64 years were 4.08 times (adjusted odds ratio (AOR): 4.08, 95% CI: 1.14–14.56) more likely to have good knowledge of LSM than their younger (⩽45 years) counterparts. Respondents who attended formal education were almost four times (AOR: 3.93, 95% CI: 1.27–12.23) more likely to have good knowledge of LSM compared to those who did not attend formal education. The study also demonstrated that respondents who were government employee and housewives were eight times (AOR: 8.06, 95% CI: 1.40–46.32) and five times (AOR: 5.10, 95% CI: 1.26–20.79) more likely to have good knowledge of LSM compared to farmers (Table 5).

**Table 5.** Bivariate and multivariate logistic regression analysis of factors associated with knowledge of LSM recommended for hypertension management among hypertensive patients at chronic care unit of HFSUH, Harar, Eastern Ethiopia, 2019 (N = 274).

Variables	Category	Knowledge		COR (95% CI)	P-value	AOR (95% CI)	P-value
		Poor (N = 37)	Good (N = 100)				
Age group	⩽45	22 (45.8%)	26 (54.2%)	1.00		1.00	
	46–64	40 (25.3%)	118 (74.7%)	2.50 (0.97–6.45)	0.059	4.08 (1.14–14.56)	0.03
	⩾65	12 (17.6%)	56 (82.4%)	3.95 (1.20–13.01)	0.024	4.90 (0.87–27.71)	0.07
Educational status	Non-educated	54 (49.0%)	56 (51.0%)	1.00		1.00	
	Educated	20 (23.8%)	144 (76.2%)	6.94 (2.98–16.19)	<0.001	3.93 (1.27–12.23)	0.02
Residence	Urban	92 (70.8%)	38 (29.2%)	3.25 (1.43–7.38)	0.005	1.967 (0.53–7.27)	0.31
	Rural	46 (63.9%)	26 (36.1%)	1.00		1.00	
Occupation	Farmer	24 (70.6%)	10 (29.4%)	1.00	**<0.001**		
	Government employee	10 (9.6%)	94 (90.4%)	22.56 (5.61–90.78)		8.06 (1.40–46.32)	0.02
	Merchant	8 (23.5%)	26 (76.5%)	7.80 (1.69–36.06)	0.009	3.49 (0.38–31.77)	0.27
	Housewife	32 (31.4%)	70 (68.6%)	5.25 (1.58–17.42)	0.007	5.10 (1.26–20.79)	0.02
Average monthly income (ETB)	⩽1000	60 (34.5%)	114 (65.5%)	1.00		1.00	
	1001–2500	8 (17.4%)	38 (82.6%)	2.50 (0.78–8.02)	0.123	2.311 (0.37–14.49)	0.37
	⩾2500	6 (11.1%)	48 (88.8%)	4.21 (1.17–15.13)	0.028	2.528 (0.40–15.94)	0.32
Duration on follow-up (year)	⩽5	64 (35.9%)	118 (64.8%)	1.00		1.00	
	>5	10 (10.9%)	82 (89.1%)	4.45 (1.60–12.37)	0.004	3.007 (0.88–10.25)	0.079

LSM: lifestyle modification; HFSUH: Hiwot Fana Specialized University Hospital; ETB: Ethiopian Birr; COR: crude odds ratio; AOR: adjusted odds ratio; CI: confidence interval.

1. 00: reference group.

In multivariate logistic regression analysis, only attitude had statistically significant association with respondents level of practice of LSM. Accordingly, respondents who had favorable attitude toward LSM recommended for hypertension management were over nine times (AOR: 9.20, 95% CI: 2.60–32.24) more likely to have good practice of LSM than those who had unfavorable attitude (Table 6).

**Table 6.** Bivariate and multivariate logistic regression analysis of factors associated with practice of LSM recommended for hypertension management among hypertensive patients at chronic care unit of HFSUH, Harar, Eastern Ethiopia, 2019 (N = 274).

Variables	Category	Practice		COR (95% CI)	P-value	AOR (95% CI)	P-value
		Poor (N = 69)	Good (N = 68)				
Educational status	Non-educated	70 (63.6%)	40 (36.4%)	1.00		1.00	
	Educated	68 (41.5%)	96 (58.5%)	2.47 (1.22–4.99)	0.012	1.149 (0.44–3.03)	0.779
Occupation	Farmer	24 (70.6%)	10 (29.4%)	1.00		1.00	
	Government employee	38 (36.5%)	66 (63.5%)	4.17 (1.27–13.65)	0.018	1.64 (0.39–6.86)	0.882
	Merchant	16 (47.1%)	18 (52.9%)	2.70 (0.66–11.09)	0.168	1.31 (0.27–6.42)	0.753
	House wife	70 (50.8%)	42 (41.2%)	1.68 (0.52–5.48)	0.390	0.92 (0.25–3.42)	0.364
Knowledge	Poor	58 (78.4%)	16 (21.0%)	1.00		1.00	
	Good	80 (40%)	120 (60.0%)	5.437 (2.26–13.10)	<0.001	1.32 (0.38–4.60)	0.661
Attitude	Unfavorable	76 (82.6%)	16 (17.4%)				
	Favorable	62 (34.1%)	120 (65.9%)	5.09 (2.33–11.13)	<0.001	9.20 (2.60–32.24)	0.001

LSM: lifestyle modification; HFSUH: Hiwot Fana Specialized University Hospital; COR: crude odds ratio; AOR: adjusted odds ratio; CI: confidence interval.

1. 00: reference group.

## Discussion

Hypertension remains as one of the most important public health challenges worldwide because of the associated morbidity, mortality and cost to the society. Despite the availability of safe and effective antihypertensive medications and the existence of clear treatment guidelines, hypertension is still inadequately controlled in a large proportion of patients worldwide. Unawareness and negative attitude toward LSM, which is important part of hypertension management, remain a major challenge.^24^ This study assessed the level of KAP of LSM and associated factors among hypertensive patients.

In the present study, 73.0% of the participants had good knowledge about LSM recommended for hypertension management. This is consistent with study conducted in Bishoftu, Ethiopia^20^ where 72.3% of the participants had good knowledge toward LSM recommended for hypertension management. However, our study finding is higher than studies conducted in Ghana,^17^ North-Western Nigeria,^18^ South-Western Nigeria,^22^ South-East Nigeria^25^ and Imo Nigeria^23^ where only 30%, 31%, 33%, 42.6% and 26.7% patients had good knowledge toward LSM recommended for hypertension management, respectively. The difference in the study finding could be due to difference in study setting, residence and educational status. Unlike our study, some studies were community based,^23,25^ some conducted on rural dwellers^23^ and the other was conducted on patients which in majority had no formal education (68.3%).^18^

Two-third (66.4%) of our study participants had favorable attitude toward LSM recommended for hypertension management. This is consistent with findings from other hospital-based study in Bishoftu, Ethiopia,^20^ Enugu, Nigeria^22^ and Turkey^26^ where more than 50% of the participants had positive attitude toward LSM. However, it is lower than finding of study in North-Western Nigeria where almost all (99%) of the study participants had positive attitude. The discrepancy might be due to the difference in attitude measurement. In our study, we used median to classify level of attitude toward LSM whereas in Nigerian study they categorized attitude as positive if respondents score between 23 and 44 and as negative if respondents score between 0 and 22 out of 44.

Regarding the overall practice level of the respondents, nearly half (49.6%) had good practice of LSM recommended for hypertension management. This finding is comparable with studies reported from South-East (42.5%) and North-West (56.7%) Nigeria.^18,25^ However, the finding is higher than study conducted in southern Ethiopia^19^ where only 27.3% of the participants had practiced recommended LSMs. The difference could be due to difference in study setting. Unlike the southern Ethiopia study which was conducted in General hospitals, the present study is conducted in specialized university hospital where health service is provided through collaborative effort of health professionals from different specialized fields. The other reason may be due to difference in level of awareness about LSM. In present study, 92.0% were aware of LSM recommended for hypertension management whereas in study conducted in south Ethiopia significant proportions (42.4%) not hear information about LSM.

In this study age, educational status and occupation of the respondents showed a significant association with knowledge of LSM in multivariate logistic regression analysis. Those respondents who were in age range of 46–64 years were 4.08 times more likely to have good knowledge of LSM than their younger (⩽45 years) counterparts (AOR: 4.08, 95% CI: 1.14–14.56, P = 0.03). This association is possibly explained as with increasing age one can learn more from experience. The other possible justification is that out of 164 (59.8%) respondents from the category of formal education, majority (58.5%) were in the age range of 46–64 years. In our study, there was no association between age ⩾65 years and knowledge of LSM. This could be due to older persons have less education, decreased cognitive function and more co-morbidities which may decrease their level of understanding.

Respondents who attended formal education were almost four times more likely to have good knowledge of LSM compared to those who did not attend formal education (AOR: 3.93, 95% CI: 1.27–12.23, P = 0.02). The association between effect of educational background and knowledge of LSM was further supported by study from Ghana.^17^ Having formal education plays a positive role in increasing the knowledge of respondents about LSM recommended for hypertension management. Educated clients would have better access to health-related information than non-educated clients and would have greater autonomy to make decision and have greater ability to use quality health care services. In other way, illiteracy may reduce the ability of clients to understand issues during counseling.

The study also demonstrated that respondents who were government employee were eight times more likely to have good knowledge of LSM compared to farmers (AOR: 8.06, 95% CI: 1.40–46.32, P = 0.02). Similarly, respondents whose occupation was housewives were five times more likely to have good knowledge of LSM compared to farmers (AOR: 5.10, 95% CI: 1.26–20.79, P = 0.02).

In the present study, only attitude had statistically significant association with level of practice of LSM in multivariate analysis. Accordingly, respondents who had favorable attitude toward LSM recommended for hypertension management were over nine times more likely to have good practice of LSM than those who had unfavorable attitude (AOR: 9.20, 95% CI: 2.60–32.24, P = 0.001). This means the better the patients had positive attitude toward LSM, the better they practiced healthy LSM. This was further supported by study conducted to assess KAP toward LSM among type 2 diabetic mellitus patients.^27^

In current study, age, educational status and monthly income did not show significant association with practice of LSM. This is in contrast to previous study conducted in southern Ethiopia where these variables showed significant association with practice of LSM.^19^ A possible explanation could be due to difference in study setting. In our study, there was no significant association between knowledge and practice of LSM. This means that being knowledgeable did not necessarily willingness to observe healthy lifestyle habits. This was also supported by another study.^27^

Although the study has many strengths, yet the study does have some limitations. First, the study is a single-center study. In addition, the data were self-report from the participants; hence, poor attitudes and practices might be denied, which affect the result of the study.

## Conclusion

The study found that levels of knowledge and attitude toward LSM recommended for hypertension management were fairly good but practice level was poor. Age range of 46–64 years, having formal education, being government employee and being housewives were factors significantly associated with good knowledge of LSM, whereas favorable attitude was the only factor significantly associated with good LSM practice. Therefore, an integrated effort from different health discipline is needed to improve patient’s KAP toward LSM for hypertension management.
